# Advancements in Artificial Intelligence for Precision Diagnosis and Treatment of Myocardial Infarction: A Comprehensive Review of Clinical Trials and Randomized Controlled Trials

**DOI:** 10.7759/cureus.60119

**Published:** 2024-05-11

**Authors:** Syed J Patel, Salma Yousuf, Jaswanth V Padala, Shruta Reddy, Pranav Saraf, Alaa Nooh, Luis Miguel A Fernandez Gutierrez, Abdirahman H Abdirahman, Rameen Tanveer, Manju Rai

**Affiliations:** 1 Internal Medicine, S Nijalingappa Medical College and Hanagal Sri Kumareshwar Hospital and Research Centre, Bagalkot, IND; 2 Public Health, Jinnah Sindh Medical University, Karachi, PAK; 3 Internal Medicine, GSL Medical College, Rajamahendravaram, IND; 4 Internal Medicine, Sri Venkata Sai Medical College and Hospital, Mahbubnagar, IND; 5 Internal Medicine, Sri Ramaswamy Memorial Medical College and Hospital, Kattankulathur, IND; 6 Internal Medicine, China Medical University, Shenyang, CHN; 7 Internal Medicine, Universidad Anahuac Queretaro Juriquilla, Queretaro, MEX; 8 Internal Medicine, Southern Medical University, Guangzhou, CHN; 9 Internal Medicine, Lakehead University, Thunder Bay, CAN; 10 Biotechnology, Shri Venkateshwara University, Gajraula, IND

**Keywords:** cardiovascular risk factors, myocardial infarction, risk assessment, machine learning, artificial intelligence, primary prevention, coronary artery disease

## Abstract

Coronary artery disease (CAD) is still a serious global health issue that has a substantial impact on death and illness rates. The goal of primary prevention strategies is to lower the risk of developing CAD. Nevertheless, current methods usually rely on simple risk assessment instruments that might overlook significant individual risk factors. This limitation highlights the need for innovative methods that can accurately assess cardiovascular risk and offer personalized preventive care. Recent advances in machine learning and artificial intelligence (AI) have opened up interesting new avenues for optimizing primary preventive efforts for CAD and improving risk prediction models. By leveraging large-scale databases and advanced computational techniques, AI has the potential to fundamentally alter how cardiovascular risk is evaluated and managed. This review looks at current randomized controlled studies and clinical trials that explore the application of AI and machine learning to improve primary preventive measures for CAD. The emphasis is on their ability to recognize and include a range of risk elements in sophisticated risk assessment models.

## Introduction and background

**Figure 1:**
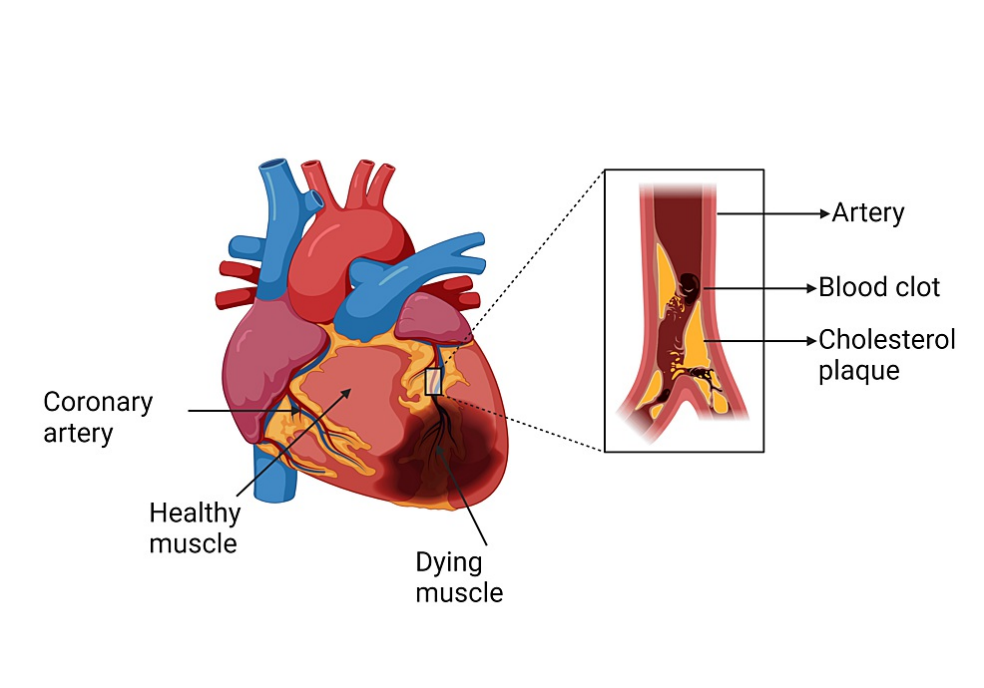
Illustration of myocardial infarction caused by acute thrombus in the coronary artery. Figure created with Biorender.com

A myocardial infarction (MI), also referred to as a heart attack, is a serious cardiovascular occurrence that is marked by an abrupt cessation of blood supply to a section of the heart muscle (Figure 1), resulting in tissue damage and possibly fatal consequences [1]. MI is a major public health concern as it is one of the top causes of death globally, requiring prompt management and precise diagnosis for the best possible patient outcomes [2].

For MI patients to be managed effectively, a precise diagnosis and choice of therapy are essential. A combination of the clinical history, physical examination, electrocardiogram (ECG) results, cardiac biomarkers (such as troponin levels), and imaging investigations (such as echocardiography and coronary angiography) is usually used to diagnose MI [3]. The ability to quickly diagnose MI lowers the risk of consequences including heart failure and arrhythmias and prevents additional damage to the heart muscle [1]. It also makes timely treatment feasible. Furthermore, the proper selection of therapy based on the unique needs of each patient is essential to maximize results and reduce side effects. Thrombolytic therapy, coronary artery bypass grafting (CABG), percutaneous coronary intervention (PCI) with stent placement, medicine (e.g., beta-blockers, statins, antiplatelet agents), lifestyle changes, and cardiac rehabilitation are possible treatments [4].

**Figure 2:**
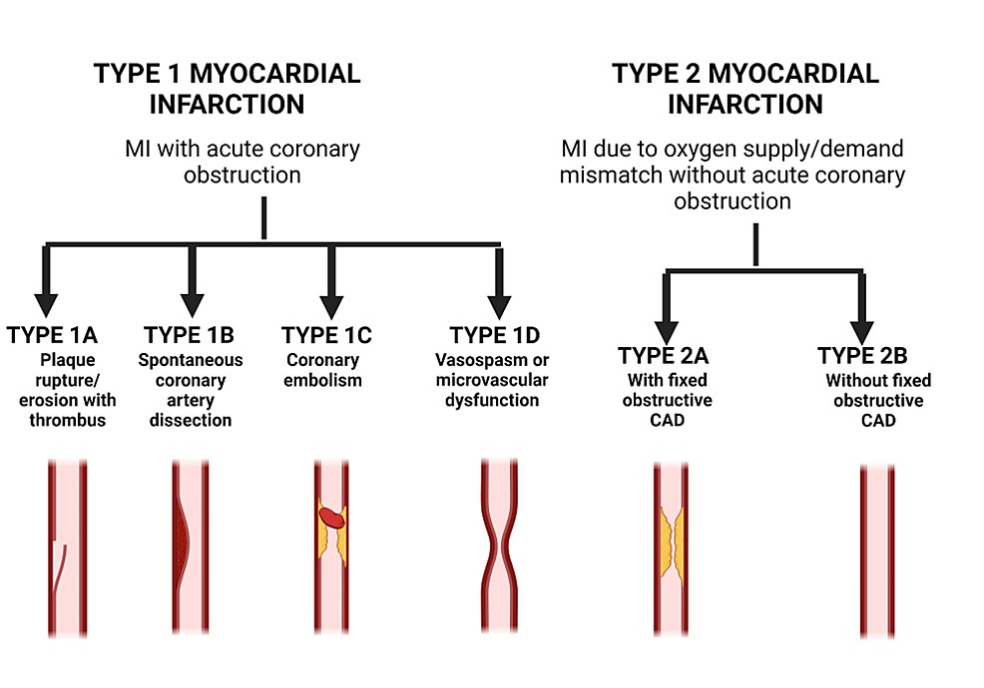
Pathophysiology of myocardial infarction. Common types of myocardial infarction (MI): Type 1 MI with acute coronary obstruction and Type 2 MI due to oxygen supply/demand mismatch without acute coronary obstruction (Figure is authors’ own creation).

Early recognition, prompt intervention, and comprehensive post-MI care are critical for optimizing outcomes and lowering the risk of recurrent events. The prognosis following MI depends on a number of factors, including the extent of myocardial damage (Figure 2), the presence of underlying comorbidities, the timeliness and effectiveness of treatment, and adherence to recommended lifestyle changes and medications [5].

With its ability to improve operational efficiency, therapeutic personalization, and diagnostic accuracy, artificial intelligence (AI) has the potential to completely transform the healthcare industry [5]. AI can analyze massive medical data sets to find trends, forecast outcomes, and help doctors make decisions by using sophisticated algorithms and machine learning (ML) techniques. AI-driven technologies have the potential to improve patient care, streamline workflows, and lower healthcare costs [6]. Examples of these innovations include image recognition for early disease identification and predictive modeling for individualized treatment regimens. Furthermore, AI-powered solutions have the potential to revolutionize the way that healthcare is delivered, allowing for proactive treatments, enhancing patient outcomes, and eventually advancing public health globally [7].

AI algorithms exhibit adeptness in examining intricate data and offer insights that surpass conventional techniques. AI improves diagnostic precision in cardiology by using sophisticated ECG and image analysis to forecast future cardiac risks. This allows for more accurate MI identification and proactive therapies [5]. Better patient outcomes are expected by the new standard of care brought about by the development of AI technology, which places an emphasis on early detection and individualized therapy [7]. AI plays a crucial role in the detection of MI by analyzing various medical data, such as ECGs and imaging scans, to identify patterns indicative of the condition. Moreover, AI aids in personalized treatment strategies by analyzing patient data to predict outcomes and recommend tailored interventions, improving patient outcomes and reducing mortality rates associated with MI. The objective of this review article is to examine various AI applications in MI diagnosis including image-based diagnosis, ECG analysis, and integration of data. Through a comprehensive analysis of existing literature and emerging trends, this review aims to elucidate the potential benefits and challenges associated with integrating AI into clinical practice for MI diagnosis. By highlighting key studies and innovations in the field, this review aims to contribute to the ongoing discourse on leveraging AI to improve patient outcomes in the management of MI.

## Review

Diagnostic tools in myocardial infarction

Clinical assessment, ECG, and biochemical markers are the main components of traditional diagnostic approaches for MI. Each has advantages and disadvantages of its own [8]. Clinical assessment includes analyzing the patient's physical examination results, medical history, and symptoms. Although the method's subjective interpretation and clinician variability can contribute to diagnostic mistakes, it can offer useful preliminary insights into the possibility of MI. Furthermore, this approach's usefulness is limited because clinical signs and symptoms of MI might be nonspecific, especially in patients with concomitant illnesses or unusual presentations [9].

Because it can identify distinctive alterations in myocardial electrical activity, ECG is a crucial tool in the diagnosis of MI. In particular, non-ST-segment elevation myocardial infarction (NSTEMI) may be suggested by ST-segment depression or T-wave inversion, whereas the presence of ST-segment elevation or new-onset left bundle branch block on ECG is suggestive of acute ST-segment elevation myocardial infarction (STEMI) [10]. ECG results, however, occasionally may not be present or may be weak, particularly in the early phases of MI or when confounding variables like bundle branch blockages or previous ST-segment abnormalities are present [11].

Myocardial damage releases biochemical markers into the bloodstream, such as cardiac troponins and creatine kinase-MB (CK-MB), which are frequently used to confirm the diagnosis of MI [12]. Elevated levels of cardiac troponins have been found within hours after the beginning of symptoms, making them extremely specific and sensitive markers of myocardial necrosis [13]. However, factors like renal failure, long-term cardiac issues, and non-cardiac sources of troponin increase may affect their diagnostic accuracy, therefore cautious interpretation is needed in clinical practice [14].

**Figure 3:**
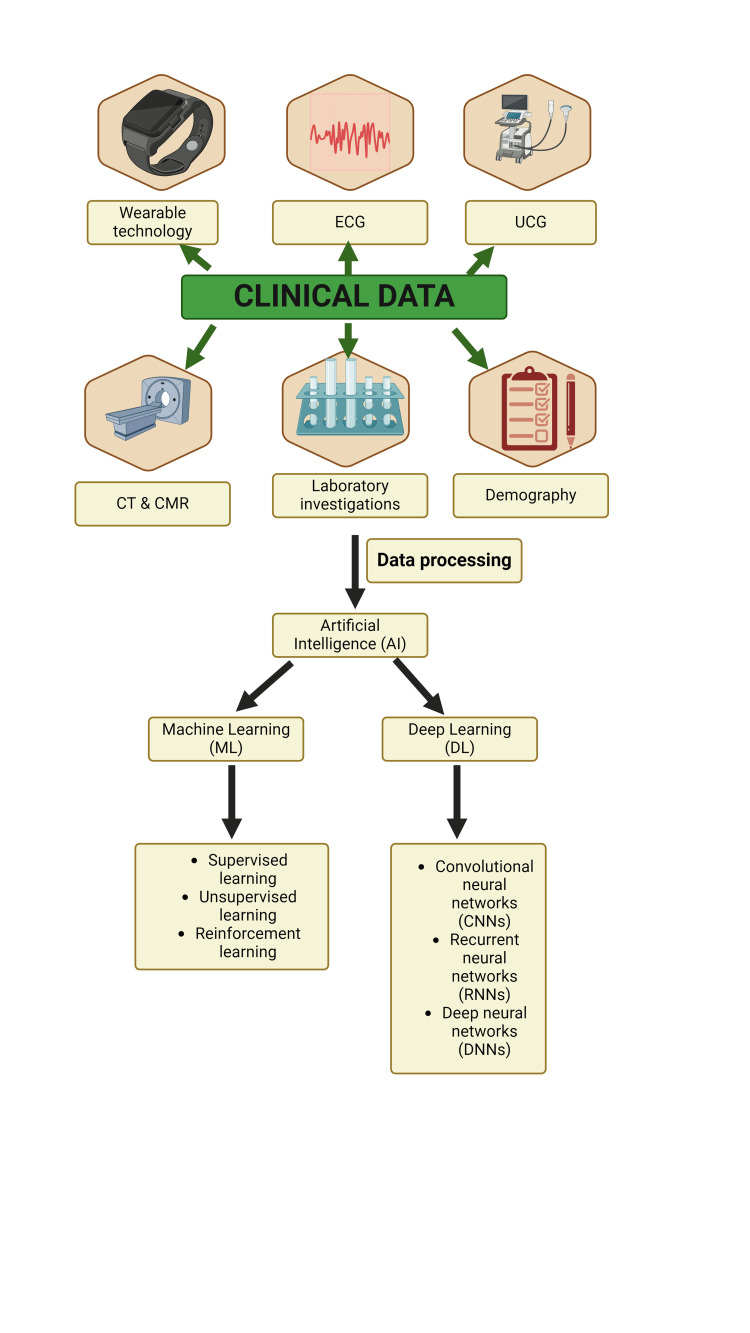
AI in CVD: Diagnostic and therapeutic perspective. ECG: Electrocardiograph; UCG: Ultrasonic cardiogram; CMR: cardiac magnetic resonance; CVD: cardiovascular disease; AI: artificial intelligence. Figure created from Biorender.com

Despite their widespread use, traditional diagnostic methods for MI have several limitations. These include variable sensitivity and specificity, reliance on subjective interpretation, potential for false-positive or false-negative results, and inability to reliably detect certain types of MI, such as non-obstructive or type 2. Modern studies underscore the importance of diagnosing cardiovascular diseases (CVD) and emphasize timely diagnosis and treatment facilitated by AI. These endeavors aim to enable MI diagnosis in remote healthcare settings, ensuring prompt primary care interventions [15]. AI employs deep learning and ML algorithms as robust analytical tools, capable of scrutinizing intricate data from imaging modalities such as stress echocardiograms (SE), cardiac magnetic resonance (CMR) imaging, and cardiac perfusion imaging (CPI). This enables earlier detection of CVDs, potentially improving patient outcomes (Figure 3).

In SE, researchers explored AI's potential for diagnosing severe coronary artery disease (CAD), utilizing ML classifiers trained to recognize specific features in SE images indicative of abnormal heart wall movement. The AI system demonstrated high accuracy in identifying severe CAD, aligning closely with physicians' observations. Moreover, when doctors integrated AI with their expertise, diagnostic confidence in severe CAD cases increased by 10%, highlighting AI's supportive role in enhancing diagnostic accuracy and confidence among clinicians [16]. Overall, these findings underscore AI's substantial impact on clinical workflows and patient care, particularly in refining patient selection for invasive procedures like coronary angiography.

CMR imaging is vital for assessing heart function and predicting future cardiac issues. Traditionally, manual analysis of CMR images is laborious and costly. However, AI is revolutionizing CMR analysis by demonstrating comparable performance to manual analysis in predicting future heart problems post-MI, particularly focusing on global longitudinal strain (GLS), a robust predictor of cardiac issues [16]. AI-powered CMR analysis has the potential to enhance assessment efficiency and cost-effectiveness significantly.

CPI involves assessing blood flow in the heart muscle using radioactive tracers. AI aids in interpreting CPI scans, with one study showing that an AI system performed on par with experienced doctors in identifying stress defects and ischemia [17]. Interestingly, AI provided not only probabilities of abnormality but also offered potential perspectives compared to traditional methods, hinting at its potential as a valuable tool for accurately detecting heart issues.

In ECG analysis, AI algorithms excel in identifying subtle abnormalities, potentially improving MI and myocardial ischemia detection. Studies suggest that AI streamlines ECG analysis, leading to faster diagnoses and better patient outcomes, even in pre-hospital settings. Deep learning algorithms trained on extensive ECG data show promise in early and accurate MI detection, with potential integration into wearable devices for continuous monitoring [9]. Another study looked at the use of serial ECG characteristics to identify acute myocardial ischemia during ambulance trips (pre-hospital phase) using an advanced AI algorithm known as Advanced Repeated Structuring and Learning Procedure (AdvRS&LP) [18]. When it came to ischemia event detection, this AI system performed better than conventional ones. Given the significance of early identification in these circumstances, AI methods such as AdvRS&LP show promise for enhancing patient outcomes and diagnostic precision. Researchers are even delving into AI on smartphones. One study examined the quantitative ECG (QCG) software, which analyzes ECG images on a smartphone. This study reported that the area under the curve of the receiver operating characteristic curve (AUC-ROC) score was significantly lower for the clinicians compared to that of the QCG score. Thus, this AI system surprisingly outperformed even highly skilled physicians in identifying an ST-elevation MI. Because paramedics can instantly identify STEMI patients, thanks to the speed and precision of QCG, emergency care could be greatly enhanced [9]. This could result in timely interventions and even save lives.

Integration of clinical data with AI enables personalized prevention plans and targeted treatments, surpassing the one-size-fits-all approach. AI analysis of large datasets informs public health strategies, aiming to mitigate CVDs globally. ML methods like MI3 and Random Survival Forests enhance risk assessment and prediction, potentially improving diagnostic accuracy and patient outcomes. Researchers looked into the MI3, a novel approach to ML [19]. This algorithm estimates a patient's risk of MI based on blood test results, age, and sex. The study found that MI3 efficiently divided patients into those with a low or high risk of MI; individuals categorized as high risk were more likely to experience an MI or die from one within a year. This suggests that MI3 could be a helpful diagnostic and risk assessment tool for medical professionals caring for people who seem to have heart problems. An alternative ML technique called random survival forests (RF) was examined in a different study [20]. This technique was used to predict various heart-related issues over a 12-year period in a sizable cohort of people who were initially clear of heart disease. The RF analysis was used to identify the critical factors, such as imaging data, ECG, and blood tests, that were important for predicting different outcomes. Notably, blood sugar levels and arterial health were important indicators of stroke and CAD, although age was the best predictor of overall death. The study's conclusion that RF performed better than current risk assessment instruments raises the possibility that it could enhance the prediction of cardiovascular risk overall.

Liu et al. developed an AI-based alarm strategy (AIS) to detect acute MI by monitoring patients' risk using chest pain, high sensitivity troponin levels, and EKG recordings [21]. The AIS aimed to assess its performance in a prospective cohort and measure the decrease in “Door to balloon time” in primary percutaneous coronary intervention. While AIS showed a low sensitivity but high specificity in diagnosing NSTEMI and STEMI, it achieved a precision and recall of 93.2% in STEMI detection, leading to a decrease in door-to-balloon time <90 min. However, false alarms and the delay in the door to cardiac catheterization laboratory activation time remained challenges. The AIS facilitated regional MI management, aiding in triaging patients and improving treatment efficiency without extensive human resources. Yet, limitations such as study scope and potential Hawthorne effects need addressing. Overall, ML algorithms offer promise as auxiliary diagnostic tools for NSTEMI, albeit with caveats regarding data accuracy and study design.

Lin et al. developed an algorithm using facial photos to detect CAD based on certain facial features associated with CAD risk factors [22]. Trained with CAD risk factors and integrated with facial photos, the algorithm achieved a sensitivity of 80% and specificity of 54%, outperforming traditional models. It didn't require additional medical history or examination, offering promise for CAD risk assessment in high-risk community populations. However, limitations include the study's focus on a Chinese population, potential selection bias, and the influence of appearance on algorithm accuracy. False-positive tests and privacy concerns also pose challenges. Further validation studies across diverse populations are warranted.

Recent studies comparing AI to traditional methods in heart diagnosis highlight AI's superiority in various aspects. ML models excel in predicting major adverse cardiovascular events (MACE) and bleeding in acute coronary syndrome (ACS) patients undergoing antithrombotic therapy, showing higher predictive ability than conventional scoring systems. According to a recent study, ML models predict MACE and bleeding better than standard scoring methods in patients with ACS using antithrombotic treatment [23]. The ML model showed better predictive power than the current scoring methods, with higher c-statistics: 0.734 vs. 0.714 for MACE and 0.670 vs. 0.671 for bleeding. These results suggest that ML has the potential to improve patient outcomes by customizing blood thinning therapy for ACS patients. Moreover, ML programs predict stent restenosis after placement more effectively than existing methods, utilizing routine medical data for prompt detection. Sampedro-Gomez et al. looked at an ML program that performed better than physicians' current techniques, Prevention of Restenosis With Tranilast and its Outcomes (PRESTO) -1, PRESTO-2, and Evaluation of Drug-Eluting Stents and Ischemic Events (EVENT), for predicting restenosis following stent implantation [24]. In order to forecast restenosis, the model discovered critical indicators such as diabetes, multiple blood artery blockages, particular procedural characteristics, and abnormal cholesterol levels. According to this research, using ordinary medical data, the new computer software could be an invaluable aid for doctors in quickly predicting stent restenosis after stent implantation. A recent study demonstrates how ML can forecast the likelihood of obstructive CAD and the necessity of angioplasty procedures to unclog blocked arteries [25]. The model outperformed the CAD2 clinical score, which is currently in use. Furthermore, irrespective of the kind of imaging test employed, the ML model correctly predicted the requirement for an operation. It took into account variables including a person's weight and the quantity and severity of artery blockages. These results demonstrate how ML might enhance heart health prediction risk. Furthermore, AI-based post-MI risk assessment, while yielding similar results to traditional methods [26]. This suggests that AI-based analysis, while not surpassing traditional methods in this instance, could be a valuable tool for streamlining the process and potentially improving efficiency in assessing post-MI cardiac risk MI. Thus, AI's ability to uncover hidden patterns, continuously learn, and automate tasks revolutionizes cardiovascular event detection, offering promising avenues for improving patient outcomes through earlier intervention.

Therapeutic decision support system

Therapy selection in MI encompasses a multifaceted approach aimed at achieving optimal patient outcomes by addressing acute ischemic injury, preventing recurrent events, and managing underlying risk factors [27]. The selection of therapy is guided by established clinical guidelines, patient-specific factors, and evolving evidence-based practices. Conventional risk assessment tools for CAD primarily rely on established risk factors such as age, gender, hypertension, diabetes, and smoking status. While these factors provide valuable insights into cardiovascular risk, they may not capture the full spectrum of individual susceptibility [28]. Chen et al. noted in their review that current primary prevention strategies often fall short in capturing the complexity of CAD risk profiles and could benefit from the integration of AI-driven approaches [29]. Therapeutic decision-making in MI has evolved with the advent of AI and ML technologies, which offer the potential to enhance clinical decision support and personalize treatment recommendations. AI-driven therapeutic decision support systems (TDSS) utilize advanced algorithms to analyze large datasets comprising clinical variables, imaging findings, biomarkers, and genetic information to generate actionable insights and facilitate evidence-based decision-making in real time [29]. These systems leverage supervised and unsupervised ML techniques to identify patterns, predict outcomes, and optimize therapeutic strategies, thereby augmenting clinician decision-making and improving patient outcomes.

**Figure 4:**
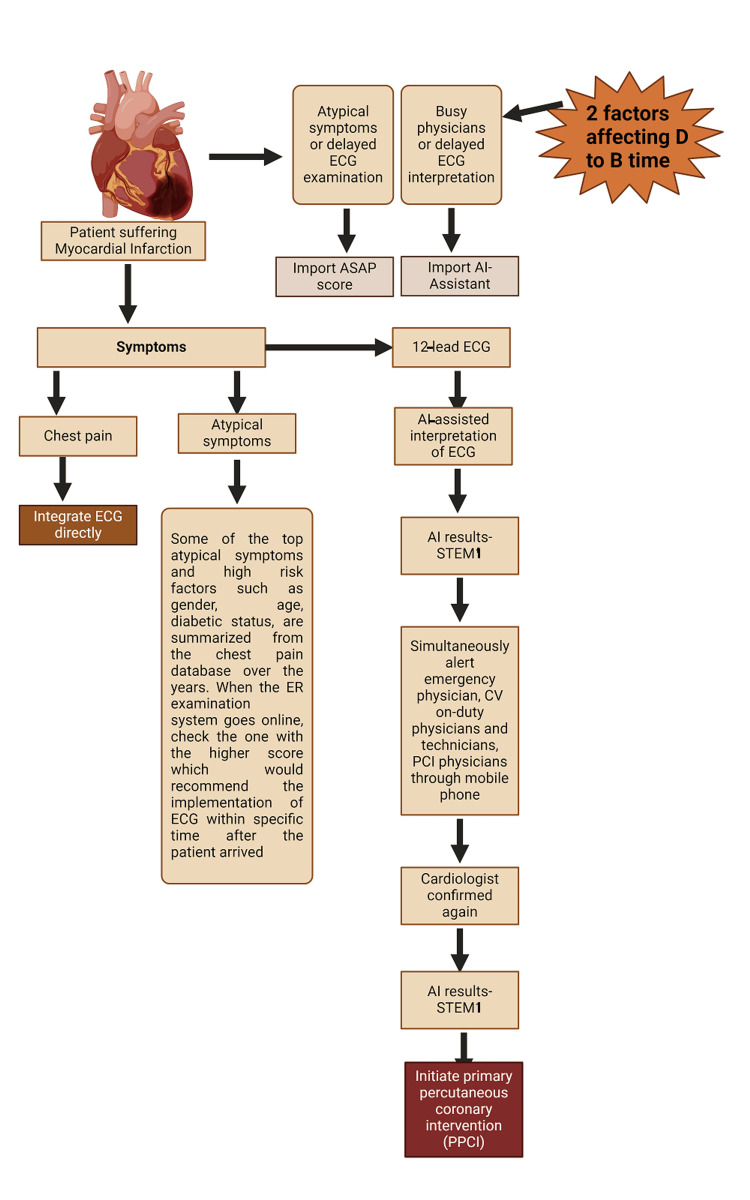
AI-assisted acute myocardial infarction decision support system. ECG: electrocardiogram; AI: artificial intelligence; ASAP score: altered consciousness, Systemic malaise, upper Abdominal pain, Past history; AI: artificial intelligence; STEMI: ST-segment elevation myocardial infarction; CV: cardiovascular. Figure created from Biorender.com

By harnessing the power of AI, researchers can combine multiple risk factors and clinical parameters to develop more nuanced and accurate risk prediction models (Figure 4). Commandeur et al. conducted a prospective study wherein ML techniques were employed to incorporate various criteria into a comprehensive risk prediction tool for MI and cardiac death [30]. Similarly, Chen et al. utilized ML algorithms for predicting intrahospital mortality in ST-elevation MI patients with type 2 diabetes mellitus [31]. Furthermore, a neural network was found to be better at predicting mortality in STEMI patients in comparison to logistic regression [32]. The results demonstrated a significant enhancement in risk prediction accuracy compared to traditional risk assessment methods, highlighting the potential of AI-driven approaches to improve primary prevention strategies for CAD.

One key application of AI in MI management is risk stratification and prognostication, wherein TDSS utilize predictive models to assess individual patient risk profiles and guide treatment intensity [33]. AI-driven risk prediction models can effectively identify high-risk patients who may benefit from aggressive therapeutic interventions, such as early revascularization or intensive pharmacotherapy while minimizing needless interventions in low-risk individuals [34]. This is achieved by integrating diverse data sources and utilizing sophisticated algorithms.

A study by Xue et al. used ML to determine which phenotypes of the lipid profile were most likely to have unfavorable outcomes after a STEMI [10]. Although the connection between dyslipidemia and unfavorable outcomes in MI is well established, traditional risk assessment techniques ignore the complex relationships between different lipoproteins and their role in raising cardiovascular risk. Thus, the study's objective was to use unsupervised ML to create a thorough lipid profile-based risk assessment tool for STEMI that is independent of previous classifications. The results of this investigation showed a statistically significant increase in the risk of all-cause mortality, rehospitalization, and cardiac rehospitalization for the phenogroup with high lipoprotein A, low HDL, and Apolipoprotein A1 levels. Based on lipid profiles, this strategy provides a logical way to identify high-risk STEMI patients, thereby enhancing risk assessment and patient outcomes.

Furthermore, by customizing therapy approaches to each patient's unique qualities, preferences, and genetic predispositions, AI-based TDSS enable tailored treatment suggestions. The myocardial-ischemic-injury-index (MII-3) algorithm measured cardiac troponin 1 in individuals suspected of having ACS in a randomized experiment. Patients who were assessed as low likelihood (17.6% vs. 1.5%)19 had a 10-fold lower rate of future MI or cardiovascular death at one year compared to those who were classified as high probability using the Hospital Anxiety and Depression Scale (HADS) 3 algorithm. Therefore, these systems continuously improve therapy algorithms based on real-world outcomes data through iterative learning and adaptation, allowing the administration of precision medicine approaches customized to each patient's own clinical profile.

In order to improve patient education on cardiovascular risk factors, Pelly et al. conducted a qualitative study in which patients who had previously experienced MI actively participated in the co-designing of an AI online app [35]. According to the study, participants strongly preferred personalized services that could provide them with feedback and guidance that was specifically tailored to them. This result is consistent with the larger healthcare trend toward patient-centered care approaches. However, addressing patient concerns along with ethical and privacy considerations is critical to the viability of adopting such individualized applications. Gelman et al. created treatment plans with AI-powered randomization strategies to address diuretic resistance in patients with congestive heart failure (CHF) [36]. Better patient response to diuretic medication was one of the study's main indicators of progress made in overcoming diuretic resistance. AI-based treatment regimens also made dosage decrease easier, which suggests better treatment outcomes and less pharmaceutical burden for patients. These results highlight how AI-driven interventions can optimize treatment plans for individuals with CHF who are resistant to diuretics.

Furthermore, by continually tracking patient reactions to therapy, modifying treatment algorithms in real-time, and projecting future clinical trajectories based on evolving clinical data, AI-driven TDSS provides dynamic treatment optimization and adaptive therapy planning. These systems provide seamless data interchange and collaborative decision-making between patients and physicians, thereby improving treatment adherence and patient involvement. They achieve this through integration with wearables, remote monitoring platforms, and electronic health records (EHRs).

Therefore, AI-based TDSS have the potential to completely transform therapeutic decision-making and customize therapy recommendations for the management of MI. AI-driven TDSS can improve clinical outcomes for MI patients by optimizing treatment selection, enhancing risk assessment, and using big data analytics and interdisciplinary collaboration. To fully achieve AI's transformational potential for MI care, more research, validation, and clinical practice integration are needed.

Challenges in AI adoption

AI holds significant promise in revolutionizing the management of MI. However, the adoption of AI in MI management presents unique challenges, ranging from technical limitations to ethical and regulatory considerations. The availability of high-quality and diverse datasets is crucial for training AI algorithms in MI diagnosis, risk stratification, and treatment optimization. Limited access to comprehensive datasets, especially longitudinal patient data, from diverse populations and healthcare settings poses challenges in developing robust AI models for MI management [37].

AI models used in MI management often operate as “black boxes,” making it challenging to interpret their decision-making processes [38]. Lack of transparency in AI algorithms can lead to mistrust among clinicians and patients, hindering their acceptance and adoption in clinical practice. Integrating AI-based decision support systems into existing clinical workflows poses challenges in terms of compatibility with EHR systems, interoperability, and usability for healthcare providers [39]. Resistance to change among healthcare professionals and concerns about AI replacing human judgment may impede the successful integration of AI into clinical practice.

Regulatory frameworks governing the use of AI in healthcare, such as data privacy and security regulations such as the Health Insurance Portability and Accountability Act (HIPAA) in the United States, pose challenges in terms of compliance and liability [40]. The lack of standardized regulations specific to AI applications in MI management may create uncertainty and hinder innovation in the field.

Hence addressing concerns regarding AI implementation in MI management is crucial. Mitigating bias in AI algorithms used for MI diagnosis and treatment recommendation is essential to ensure fairness and equity across diverse patient populations. Rigorous validation and testing of AI models on diverse datasets, along with the implementation of fairness-aware AI techniques, can help address bias concerns.

Enhancing the interpretability and explainability of AI models is critical to gaining clinicians trust and facilitating their adoption in clinical practice [38]. Explainable AI (XAI) techniques, such as model-agnostic methods and feature attribution algorithms, can provide insights into AI model predictions and decision-making processes [41]. Protecting patient privacy and ensuring data security are paramount when implementing AI-based systems for MI management [15]. Adherence to data protection regulations, encryption of sensitive health information, and robust cybersecurity measures are essential to safeguard patient data [42].

Considering regulatory considerations and ethical implications, regulatory agencies must develop clear guidelines and standards for the development, validation, and deployment of AI-based tools in MI management. Collaboration between regulatory bodies, healthcare providers, AI developers, and patient advocacy groups is crucial to address regulatory challenges and ensure patient safety and quality of care.

Ethical considerations, such as transparency, accountability, beneficence, and non-maleficence, should guide the development and implementation of AI technologies in MI management. Stakeholders must adhere to ethical guidelines and principles, prioritize patient autonomy and well-being, and mitigate potential risks associated with AI adoption in clinical practice [43].

Continued research and development in AI algorithms, including deep learning, natural language processing, and reinforcement learning, can enhance the accuracy and performance of AI models for MI diagnosis, risk prediction, and treatment optimization. Integration of AI with emerging technologies, such as wearable devices, remote monitoring systems, and point-of-care diagnostics, holds promise for real-time MI detection and personalized treatment delivery [44]. Collaboration between academia, industry, and healthcare institutions is essential to address the technical, regulatory, and ethical challenges in AI adoption for MI management. Multidisciplinary research initiatives focusing on data sharing, model validation, and clinical validation can accelerate the translation of AI technologies from research to clinical practice. Table 1 summarizes some of the clinical studies conducted on the use of AI in MI.

**Table 1:** Studies on the utilization of artificial intelligence (AI) in myocardial infarction. ML: machine learning; STEMI: ST-elevation myocardial infarction; CAD: coronary artery disease

S. No.	Study	Description	Merits of the Study
1.	Choi et al. [9]	Compares the diagnostic performance of artificial intelligence with physicians in interpreting ECG images for ST-elevation myocardial infarction (STEMI)	In STEMI diagnosis, an image-based AI system can perform better than physicians, and its efficacy was unaffected by changes in the settings surrounding picture acquisition.
2.	Xue et al. [10]	Investigates the use of ML based on lipid profiles for risk stratification in STEMI patients	Statin-modified cardiovascular risks are likely to have a negative clinical result for STEMI patients with elevated Lp(a) and decreased HDL-C and apoA1 concentrations.
3.	Backhaus et al. [16]	Investigates AI's role in automated myocardial strain quantification for risk stratification post-MI	Automation may increase productivity and support the implementation of clinical routines, given the high agreement of automated global longitudinal strain (GLS) and the similarly high accuracy of risk prediction when compared to the reference standard of manual studies.
4.	Nakajima et al. [17]	Evaluates the diagnostic performance of artificial neural networks (ANN) detecting ischemia using myocardial perfusion imaging (MPI)	While ANN's diagnostic performance was comparable to traditional scoring techniques, it could offer an alternative perspective on abnormality, making it a viable approach to assess abnormality in MPI.
5.	Sbrollini et al. [18]	Developed an Advanced Repeated Structuring and Learning Procedure (AdvRS&LP) for detecting acute myocardial ischemia using serial 12-lead ECGs	Neural networks (NNs) outperformed the Glasgow program (Uni-G) algorithm and the logistic regression technique in testing, with a statistically significant (P-value less than 0.05) difference. The affirmative outcomes highlight the significance of serial ECG comparison in ischemia identification, and the NNs generated by AdvRS&LP appear to be dependable instruments concerning clinical application and generalization.
6.	Doudesis et al. [19]	Validated a ML algorithm (Myocardial-Ischemic-Injury-Index- M13) for MI diagnosis in diverse populations	The MI3 algorithm correctly predicted serious cardiovascular events and calculated the probability of MI in consecutive individuals having serial cardiac troponin testing for suspected acute coronary syndrome. The MI3 method could enhance the diagnosis and risk assessment of individuals with suspected acute coronary syndrome by offering individual probabilities.
7.	Ambale-Venkatesh et al. [20]	Examined ML-based cardiovascular event prediction using data from the Multi-Ethnic Study of Atherosclerosis	In a population that was previously asymptomatic, ML combined with deep phenotyping increases prediction accuracy in cardiovascular event prediction. Without making any apriori assumptions about causality, these techniques might provide a further understanding of subclinical illness indicators.
8.	Liu et al. [21]	Investigated an AI-based alarm strategy for acute MI management, enhancing clinical decision-making	AI-based alarm strategy reduces ECG-to-cardiac catheterization laboratory activation (EtoCCLA) and door-to-balloon (DtoB) times significantly and facilitates primary percutaneous coronary intervention (PPCI) by providing front-line physicians with a prompt and dependable diagnostic decision support system.
9.	Lin et al. [22]	Explored the feasibility of using deep learning with facial photos to detect coronary artery disease	This method may be useful for community-based CAD screening or pre-test CAD likelihood estimation in outpatient clinics.
10.	Gibson et al. [23]	Compared ML with traditional methods for risk stratification in acute coronary syndrome	In comparison to conventional risk stratification techniques, the super learner algorithm obtained the greatest c-statistic for major adverse cardiovascular events (MACE) prediction and was well calibrated on both efficacy and safety outcomes. This analysis shows how ML is being used in the modern era to inform treatment choices for antithrombotic medication at the patient level.
11.	Sampedro-Gomez [24]	This study aimed to utilize ML to differentiate stent restenosis (SR) from the current prediction scores of SR. In order to create a practical model, researchers made predictions using only the data that are typically collected in daily practice, without incorporating any other factors.	Applied immediately after stent implantation, an ML model better differentiates those patients who will present with SR over current discriminators.
12.	Baskaran et al. [25]	Investigated imaging and clinical variables using ML for predicting obstructive CAD and revascularization	The ML model fared better than CAD2 for obstructive CAD. BMI is a significant factor. The imaging variables in this ML model had the strongest correlation with revascularization. Model performance was not influenced by the imaging modalities. Model performance was decreased when imaging variables were removed.
13.	Schuster et al. [26]	Developed a fully automated cardiac assessment system post-MI for diagnostic and prognostic stratification	In individuals who have had an MI, user-independent volumetric assessments using completely automated software are feasible and yield results that are equally predictive of significant adverse cardiac events when compared to conventional analyses.
14.	Commandeur et al. [30]	The study assessed the effectiveness of ML in predicting the long-term risk of MI and cardiac death in asymptomatic individuals. This was done by combining clinical characteristics with measurements of coronary artery calcium (CAC) and automated quantification of epicardial adipose tissue (EAT).	The study discovered that the utilization of ML to incorporate clinical and quantitative imaging-based characteristics substantially enhances the ability to predict MI and cardiac death in comparison to the conventional clinical risk assessment.
15.	Chen et al. [31]	The study aimed to assess the predictive performance of ML models in determining the in-hospital mortality rate among patients with STEMI and type 2 diabetes mellitus (T2DM). The study conducted a comparison of six ML models, namely random forest (RF), CatBoost classifier (CatBoost), naïve Bayes (NB), Extreme Gradient Boosting (XGBoost), gradient boosting classifier (GBC), and logistic regression (LR), using the Global Registry of Acute Coronary Events (GRACE) risk score.	Among all models, the CatBoost model demonstrated the best predictive performance. A ML algorithm, such as the CatBoost model, may prove clinically beneficial and assist clinicians in tailoring precise management of STEMI patients and predicting in-hospital mortality complicated by T2DM.
16.	Niedziela et al. [32]	The objective of the study was to ascertain the superiority of an NN over LR in predicting mortality in patients with STEMI	Both NN and LR had good predictive values. Better results were obtained in the NN approach regarding the statistical quality of the models - AUROC 0.8422 vs. 0.8137 for LR.
17.	Pelly et al. [35]	Investigated the viewpoints of individuals who have experienced MI and healthcare experts regarding the utilization of artificial intelligence for the purpose of preventing subsequent occurrences of MI. A total of 38 participants, consisting of 22 with a history of MI and 16 health professionals, took part in three rounds of focus groups conducted using videoconferencing.	The results offer valuable perspectives from end-users to enhance the chances of effectively implementing and adopting AI-enabled technologies in the field of medical imaging, which can be applied to other areas of chronic disease management as well.

## Conclusions

The adoption of AI in MI management holds immense potential to improve patient outcomes, enhance clinical decision-making, and optimize healthcare delivery. However, addressing the challenges, concerns, and ethical implications associated with AI implementation is essential to ensure the responsible and equitable use of AI technologies in clinical practice. By leveraging collaborative research efforts, regulatory frameworks, and ethical guidelines, stakeholders can navigate the complexities of AI adoption and realize the transformative impact of AI on MI management.

In the realm of MI diagnosis, AI excels in image-based analysis, ECG interpretation, and integration of diverse clinical data. From detecting subtle abnormalities indicative of ischemic injury to predicting future cardiac risks, AI algorithms demonstrate superior performance compared to traditional methods, enhancing diagnostic accuracy and enabling preemptive interventions. Furthermore, by continuously tracking patient responses to therapy and modifying treatment algorithms in real-time, AI-driven decision support systems enable dynamic treatment optimization, guaranteeing individualized and efficient care delivery.

The integration of AI in MI management poses certain obstacles that need to be overcome, even with its promising revolutionary capabilities. Widespread adoption is hampered by technical issues, such as the lack of high-quality datasets and the interpretability of AI models. To guarantee patient safety and regulatory compliance, ethical and regulatory factors including data privacy, transparency, and liability need to be carefully taken into account. Furthermore, building clinician trust and easing the adoption of AI algorithms into clinical practice require eliminating bias in AI systems and improving their interpretability.

Hence, in order to overcome these obstacles and fully utilize AI in MI management, cooperation among stakeholders, including regulatory agencies, healthcare providers, AI developers, and patient advocacy groups, is crucial. AI has the power to completely change the field of cardiovascular medicine by emphasizing patient-centric treatment, abiding by ethical standards, and stimulating creativity through interdisciplinary research projects. This will open the door to better patient outcomes and increase public health worldwide.
